# Investigating the Role of *FoxP3* in Renal Cell Carcinoma Metastasis with *BAP1* or *SEDT2* Mutation

**DOI:** 10.3390/ijms241512301

**Published:** 2023-08-01

**Authors:** Shan Xu, Xinfeng Hu, Yue Chong, Guodong Zhu

**Affiliations:** 1 Department of Urology, The First Affiliated Hospital of Xi’an Jiaotong University, Xi’an 710061, China; 2 Oncology Research Laboratory, Key Laboratory of Environment and Genes Related to Diseases, Ministry of Education, Xi’an 710061, China; 3 Key Laboratory for Tumor Precision Medicine of Shaanxi Province, Xi’an Jiaotong University, Xi’an 710061, China

**Keywords:** renal cell carcinoma, *FoxP3*, *BAP1*, *SEDT2*, metastasis

## Abstract

Forkhead box protein P3 (*FoxP3*) primarily functions as the master regulator in regulatory T cells (Tregs) differentiation, but its high level of expression has also been found in tumor cells recently. The aim of our study was to clarify the role of *FoxP3* in renal cell carcinoma (RCC) progression and metastasis. We verified the *FoxP3* characteristic clinicopathological data from The Cancer Genome Atlas (TCGA) database using bioinformatics tools. Meanwhile, RNA sequencing was performed to determine the *FoxP3* biofunction in RCC progression. Our results showed that high expression of *FoxP3* was found in *BAP1*- or *SETD2*-mutant patients with RCC, and a higher *FoxP3* expression was related to worse prognosis. However, there was no statistically significant relationship between the *FoxP3* IHC score and RCC malignant progression owning to the limited number of patients in our tissue microarray. Using in vitro *FoxP3* loss-of-function assays, we verified that silencing *FoxP3* in 786-O and ACHN cells could inhibit the cell migration/invasion capability, which was consistent with the data from RNA sequencing in 786-O cells and from the TCGA datasets. Using an in vivo nude mice orthotopic kidney cancer model, we found that silencing *FoxP3* could inhibit tumor growth. In conclusion, our study demonstrated that *BAP1* or *SEDT2* mutation could lead to higher expression of *FoxP3* in RCC patients, and *FoxP3* could eventually stimulate RCC cells’ invasion and metastasis, which might indicate that *FoxP3* could function as a potential oncogene in RCC progression.

## 1. Introduction

Kidney cancer is the 14th most common global malignancy, with an estimated 431,288 new cases in 2020 [1]. Renal cell carcinoma (RCC) is the most common type of kidney cancer, accounting for nearly 3% of all cancers worldwide [2]. In recent years, the incidence of RCC has rapidly increased worldwide, including in China [3]. According to the World Health Organization’s report, there were approximately 79,000 new cases of kidney and renal pelvis cancer in the United States in 2022. It has been found that the incidence of RCC increases exponentially with age and is approximately twice as common in men as in women [4]. Generally, clear-cell renal cell carcinoma (ccRCC) accounts for approximately 75% of RCC cases [5], and papillary RCC is the second most common tumor [6]. RCC is known to be radio-resistant and chemo-resistant, and the outcome of metastatic RCC is very poor [5,7,8]. Up to 17% of patients have distant metastases at the time of diagnosis [9,10]. In 2020, 179,368 people died from RCC globally [1]. For patients with stage III RCC, the five-year survival rate is approximately 60%; furthermore, the five-year survival rate for patients with stage IV RCC is less than 10% [6,11]. In recent years, some specific genes have been demonstrated to be associated with the development and progression of RCC. Chromosomal genetic disorders, such as von Hippel Lindau (*VHL*) syndrome with *VHL* gene mutation and hereditary leiomyomatosis and renal cell cancer with fumarate hydratase gene (*FH*) mutation, have been shown to be associated with the development of RCC [12]. It has also been investigated that patients with mutations in certain genes, such as BRCA1 associated protein 1 (*BAP1*) and succinate dehydrogenase complex subunit B (*SDHB*), are also at a higher risk for developing RCC [13].

Regulatory T cells (Tregs) are an immunosuppressive subpopulation of CD4^+^ T cells and account for approximately 5–10% of peripheral CD4^+^ T cells in the blood of healthy individuals [14]. Tregs are characterized by the expression of the master transcription factor forkhead box protein P3 (*FoxP3*) [15,16], which is a core regulator for Tregs function [17], and *FoxP3* can also regulate the expression of several related genes, such as *CD25* and *IL-2* [18]. Tregs can suppress abnormal/overactive immune responses to auto- and non-self-antigens [19]. It has been demonstrated that RCC contains abundant infiltrating lymphocytes [20]. Additionally, some reports have suggested that lymphocytes within RCC tumors were less functional [21], which might be related to the immunosuppression of Tregs regulated by *FoxP3*. Indeed, investigators have found an increased number of Tregs in RCC [22], and those increased Tregs might be associated with poor overall survival (OS) and progress-free survival (PFS) [23,24]. In humans, it has been shown that the tumor-infiltrating Tregs can be found in cancer cells that originate from the head, neck, chest, lung, liver, gastrointestinal tract, pancreas, and ovaries [25], and their abundant presence is usually associated with poor clinical prognosis. High infiltration of Tregs is associated with poor survival in various cancer patients [19,26,27,28]. Therefore, the exhaustion of Tregs and the control of Tregs function by modification of *FoxP3* might be an effective strategy to increase antitumor immune responses.

*FoxP3* is a specific biomarker of Tregs that can infiltrate into the microenvironment of many tumor types, and it is related to poor prognosis for cancer patients [26,27,28]. In Tregs, *FoxP3* acts as a chromatin remodeler by interacting with enhancer of zeste homologue 2 (*EZH2*), histone deacetylase 1 (*HDAC1*), *P300*, etc. *FoxP3* acts as a trans-factor repressor through its interaction with yin yang 1 (*YY1*), runt-related transcription factor 1 (*RunX1*), forkhead box protein1 (*FoxP1*), etc. *FoxP3* also acts as a trans-factor activator despite interacting with signal transducer and activator of transcription 3 (*STAT3*), *EOS* (a zinc finger transcription factor that belongs to the Ikaros family), RNA polymerase II, etc. Accumulating evidence indicates the role of *FoxP3* in the phenotypic stability, metabolic fitness, and regulatory function of Tregs and the mechanism of immune dysregulation [29]. There is increasing evidence that the expression of *FoxP3* can also be detected in tumor cells. *FoxP3* was first reported in human pancreatic cancer cells, and later, it was also detected in breast cancer, prostate cancer, non-small cell lung cancer (NSCLC), gastric cancer, thyroid cancer, melanoma, and hepatocellular carcinoma [30,31,32,33]. According to published reports, there is still controversy regarding the biofunction of *FoxP3* in different types of cancer. For instance, in breast cancer, *CD44* could induce the expression of *FoxP3*, which could inhibit angiogenesis by down-regulating vascular endothelial growth factor (*VEGF*), and *FoxP3* could also induce apoptosis by controlling a miR-146/*NF-κB* negative feedback loop [34]. The literature has indicated that *FoxP3* is a tumor suppressor in breast, prostate, and gastric cancers [31,34,35]. Nevertheless, the expression of *FoxP3* was found to be associated with the degree of gastric cancer differentiation, and it can promote gastric cancer proliferation, migration, and invasion through the *TGF-β* pathway [35]. In large-cell lung cancer, *FoxP3* could promote the tumor growth and metastasis by activating the *Wnt*/*β-catenin* signaling pathways [36]. Additionally, it was found that *FoxP3* had a high expression in cervical cancer and could promote the cancer cells’ metastasis through immune escape [25]. In kidney cancer, Inamura K et al. reported that the immune checkpoint protein B7-H3 expressed in ccRCC might interact with *FoxP3*-positive Tregs in tumors and suppress their immunity [33]. Furthermore, *FoxP3*-positive cells were detected to be accumulated at the border between malignant and adjacent benign kidney tissues [37]. To the best of our knowledge, whether *FoxP3* plays a key role as a tumor suppressor gene or an oncogene in RCC has not been demonstrated.

In this study, we performed gain- and loss-of-function assays of *FoxP3* to explore its biofunction in RCC progression. Additionally, we performed immunohistochemistry assays to confirm whether *FoxP3* is associated with the clinicopathological features of RCC and designed a nude mice orthotopic RCC model to verify *FoxP3* function in vivo. Our preliminary data might suggest that *FoxP3* could modulate the RCC tumor microenvironment and promote RCC aggressiveness.

## 2. Results

### 2.1. FoxP3 Was Oncogenic and Correlated with Worse Clinical Outcomes

**Figure 1:**
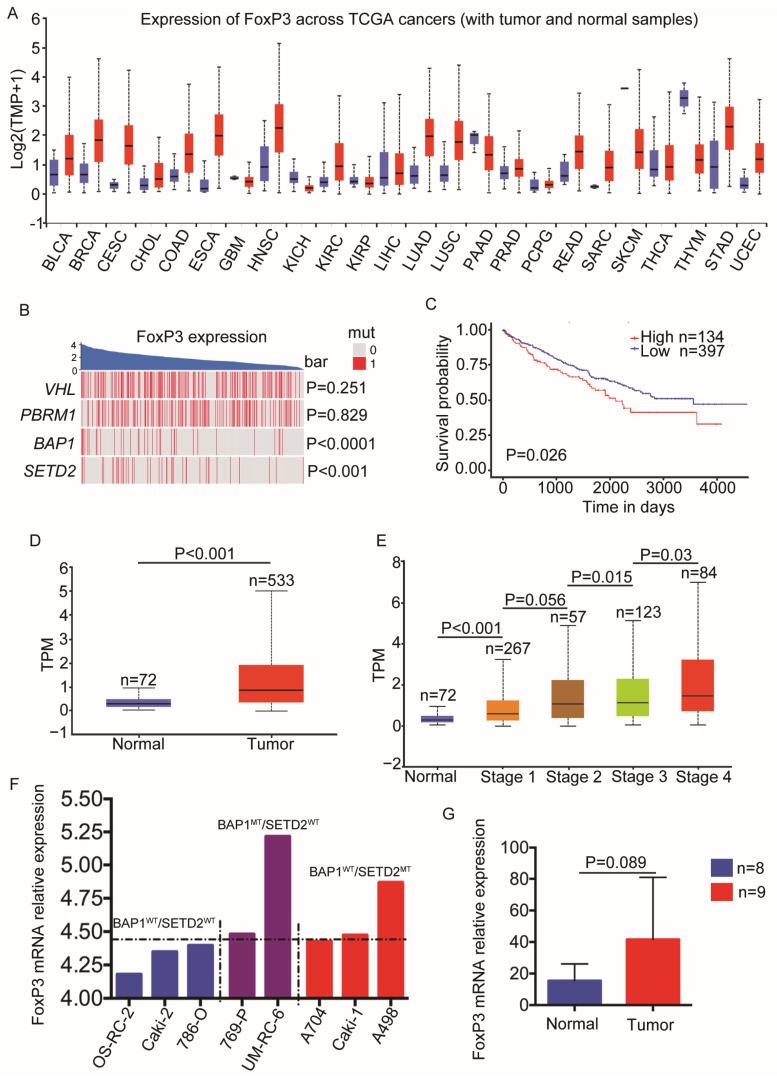
*FoxP3* expression in ccRCC was positively correlated with mutations in *BAP1* or *SETD2*, and high *FoxP3* expression level in ccRCC was associated with higher tumor stage and worse survival. (**A**) Box plots showing the expression of *FoxP3* mRNA level in 23 different cancer types across TCGA cancers (with tumor and normal samples). The darker horizontal line within the box represents the median value of *FoxP3* expression. The lower and upper limits of the box represent the 1st and 3rd quartiles of *FoxP3* expression. Whiskers indicate the maximum or minimum values of *FoxP3* expression. BLCA, bladder urothelial carcinoma; BRCA, breast invasive carcinoma; CESC, cervical squamous cell carcinoma and endocervical adenocarcinoma; CHOL, cholangiocarcinoma; COAD, cholangiocarcinoma; ESCA, esophageal carcinoma; GBM, glioblastoma multiforme; HNSC, head and neck squamous cell carcinoma; KICH, kidney chromophobe; KIRC, kidney renal clear cell carcinoma; KIRP, kidney renal papillary cell carcinoma; LIHC, liver hepatocellular carcinoma; LUAD, lung adenocarcinoma; LUSC, lung squamous cell carcinoma; PAAD, pancreatic adenocarcinoma; PRAD, prostate adenocarcinoma; PCPG, pheochromocytoma and paraganglioma; READ, rectum adenocarcinoma; SARC, sarcoma; SKCM, skin cutaneous melanoma; THCA, thyroid carcinoma; THYM, thymoma; STAD, stomach adenocarcinoma; UCEC, uterine corpus endometrial carcinoma. (**B**) Distribution of *FoxP3* expression level in ccRCC with mutations in *VHL*, *PBRM1*, *BAP1*, and *SETD2*. The blue part shows the expression level of *FoxP3* ranked from high to low. The distribution below shows whether the four genes are mutated at the corresponding *FoxP3* expression levels. The red vertical line indicates that the gene was mutated, and the grey vertical line indicates that the gene was not mutated. The *p*-values on the right side indicate the statistical significance in the distribution of the gene, respectively. (**C**) The line graph shows the change in survival rate of ccRCC with high versus low *FoxP3* expression levels at different times. The red fold line shows the change in survival over time for 134 ccRCC patients with a high *FoxP3* expression level. The blue line represents the change in survival over time in 397 ccRCC patients with low *FoxP3* expression. (**D**) Box plots showing the *FoxP3* expression level in ccRCC tissues versus normal renal tissues. The red part shows the *FoxP3* expression level in 533 ccRCC cases, and the blue part shows the *FoxP3* expression level in 72 normal kidney tissues (TPM: transcript per million). (**E**) Box plots showing the *FoxP3* expression level in normal (72), stage Ⅰ (267), Ⅱ (57), Ⅲ (123), and Ⅳ (84) of ccRCC in the TCGA database, respectively. (**F**) Bar graph showing the difference in *FoxP3* mRNA expression in different ccRCC cell lines. The blue part shows the three ccRCC cell lines with wild type *BAP1* and *SETD2* (*BAP1*^wt^/*SETD2*^wt^). The purple section shows two ccRCC cell lines with only *BAP1* mutation (*BAP1*^mt^/*SETD2*^wt^). The red part shows 3 ccRCC cell lines with only *SETD2* mutation (*BAP1*^wt^/*SETD2*^mt^). (**G**) Histogram showing the mRNA expression level of *FoxP3* in ccRCC versus normal kidney tissues in the GSE781 dataset. The red part shows 9 cases of ccRCC, and the blue part shows 8 cases of normal kidney tissues. The upper limits of the box represent the 3rd quartiles of *FoxP3* expression. Whiskers indicate the maximum values of *FoxP3* expression.

To explore and confirm the transcription level of *FoxP3* in ccRCC, we analyzed the *FoxP3* mRNA level in 23 different cancer types. As shown in Figure 1A, the transcription level of *FoxP3* was compared with adjacent normal tissues from cancer patients, including ccRCC. Through analyzing the TCGA transcriptome and mutational data of ccRCC patients, we found that the *FoxP3* mRNA level was significantly and positively correlated with mutations in the *SET* domain containing 2 (*SETD2*; *p* < 0.0001) and *BRCA1* associated protein 1 (*BAP1*; *p* < 0.001) genes (Figure 1B). We predicted that *FoxP3* was dysregulated in ccRCC patients and might be associated with the clinicopathological factors and prognosis.

To investigate the role of *FoxP3* in ccRCC clinical outcomes, the patients’ overall survival (OS) was obtained in UALCAN for analyzing cancer OMICS data. As shown in Figure 1C, a higher *FoxP3* expression was related to a worse outcome. Subsequently, we found that *FoxP3* expression was associated with a high stage (Figure 1D,E). We verified the *SETD2* and *BAP1* mutations and the *FoxP3* expression in ccRCC cell lines. Consistent with the results showed in Figure 1B, the FoxP3 expression level was much higher in the *BAP1* mutation (769-P and UM-RC-6) and *SETD2* mutation RCC cell lines (A704, Caki-1, and A498) than their specific wild-type cell lines (OS-RC-2, Caki-2, and 786-O) (Figure 1F). A higher FoxP3 expression level in tumor tissues than in benign tissues was also validated in the GSE781 datasets, which included nine tumor and eight benign tissues (Figure 1G).

**Figure 2:**
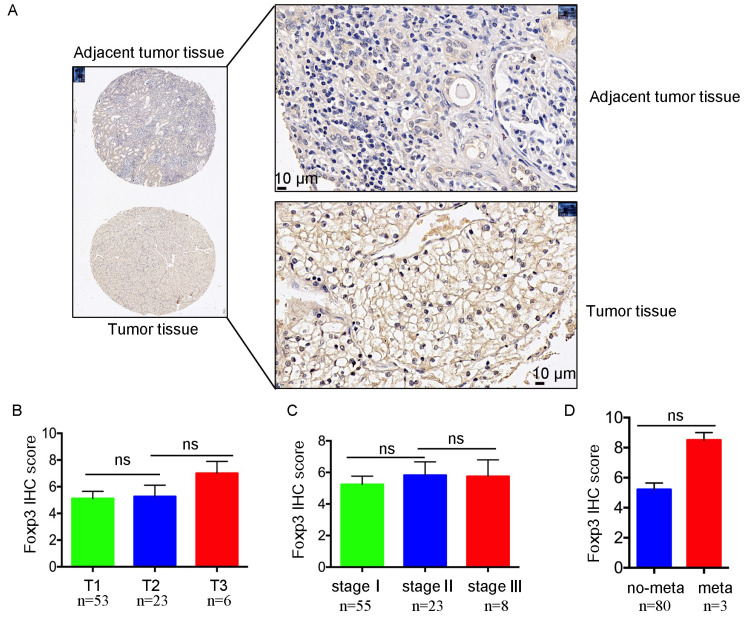
In ccRCC tissue microarray, *FoxP3* was highly expressed in the tumor and might correlate with ccRCC stages. (**A**) Representative images of adjacent normal kidney tissues (upper panel) and kidney tissues (lower panel) after detection by IHC. (**B**) Histogram showing the *FoxP3* average IHC score in grade T1 (53), T2 (23), and T3 (6) of ccRCC in the tissue microarray, respectively. IHC staining was graded as follows: 0 for 0%, 1 for ≤25%, 2 for 25–50%, 3 for 50–75%, and 4 for ≥75%. The IHC intensity was scored as follows: 0 for no staining, 1 for weakly positive staining, 2 for moderately positive staining, and 3 for strongly positive staining (ns: no statistical significance). (**C**) Histogram showing the *FoxP3* average IHC score in stage Ⅰ (55), Ⅱ (23), and Ⅲ (8) of ccRCC in the tissue microarray, respectively. (**D**) Histogram representing the *FoxP3* average IHC score in metastatic and non-metastatic ccRCC in tissue microarray, respectively (meta: metastasis occurred; no-meta: no metastasis occurred).

To further validate the relationship between *FoxP3* expression level and the patients’ clinicopathological characteristics, we performed *FoxP3* immunohistochemistry (IHC) in a ccRCC tissue microarray, including 90 tumor tissues and their paired 90 adjacent normal kidney tissues. The IHC results demonstrated that the *FoxP3* in tumor cell protein expression was higher in tumors than in their adjacent normal kidney tissues (Figure 2A), and a higher expression level of *FoxP3* protein in tumor cells was related to higher TNM stages (Figure 2B–D). However, the expression level of *FoxP3* in tumor cells had no significant differences between different tumor grades in our 90 samples. Collectively, these data suggested that the expression level of *FoxP3* was higher in tumor tissues, and that it might be positivity correlated with the ccRCC stages.

### 2.2. FoxP3 Could Facilitate RCC Tumor Metastasis

**Figure 3:**
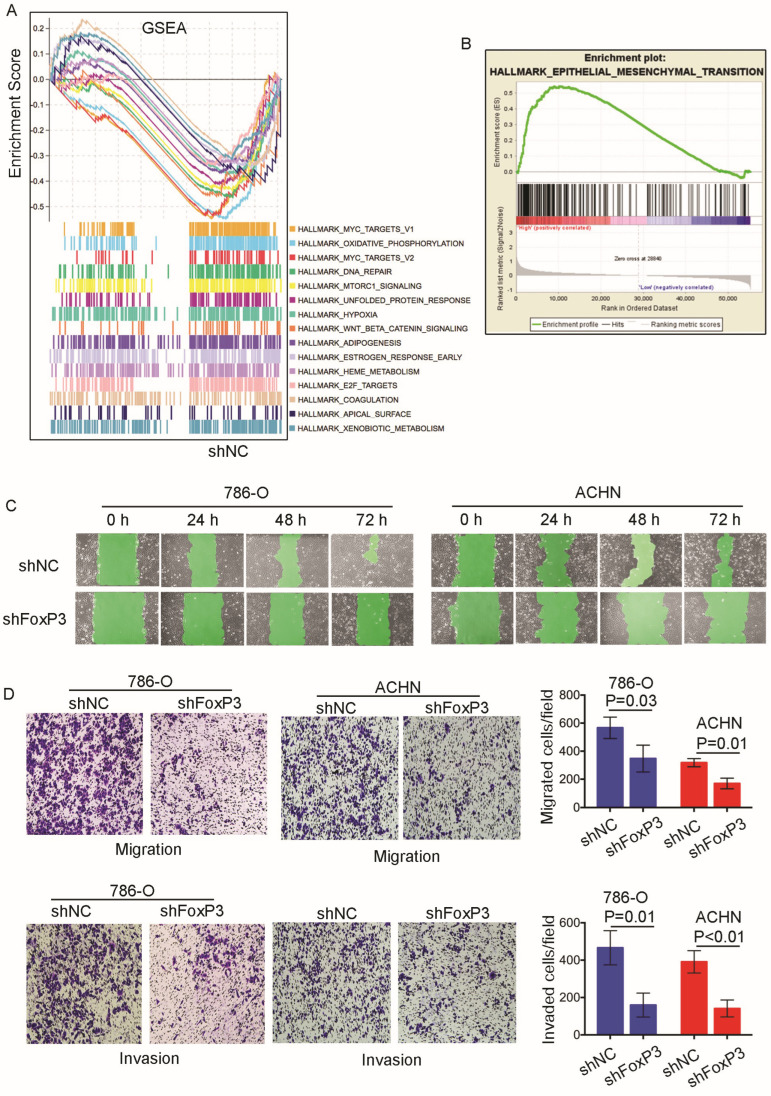
High expression of *FoxP3* could promote migration, invasion, and metastasis of ccRCC in vitro, and it might be associated with epithelial–mesenchymal transition (EMT). (**A**) Gene Set Enrichment Analysis (GSEA) images showing the difference in the expression of tumor-associated pathways between *FoxP3*-silenced 786-O cells and control cells (sh*FoxP3*: *FoxP3*-silenced 786-O cells; shNC: normal control 786-O cells). Each colored line segment in the figure corresponds to the pathway of the corresponding color on the right side, respectively. The vertical lines in the lower panel represent each gene in the specific pathway, respectively. The horizontal lines of the upper graph represent the genes at the corresponding positions in the lower graph, and the vertical lines represent the enrichment score (ES). The peaks of its vertical line coordinate the ES value of the gene set. The peak appearing at the front end of the sequenced gene set (ES value > 0) indicates that the pathway was up-regulated, and the peak appearing at the back end (ES value < 0) indicates that the pathway was down-regulated. (**B**) The Gene Set Enrichment Analysis (GSEA) image showing the difference in expression of the “Hallmark_epithelia_mensenchymal_transition_targets” pathway between the *FoxP3* high-expression group and the *FoxP3* low-expression group. The top part of the graph was described previously. The red part in the middle of the graph indicates that the gene was highly expressed in the *FoxP3* high-expression group. The blue part indicates that the gene was highly expressed in the *FoxP3* low-expression group. The lower part of the graph shows the rank in the ordered dataset, where a value greater than 0 means that the gene was more highly expressed in the *FoxP3* high-expression group. The lower the vertical line is above 0, the higher the expression of the corresponding gene in the *FoxP3* low-expression group. (**C**) The images of the wound healing assay illustrate the difference in cell migration ability between the *FoxP3*-silenced and control groups of 786-O cells and ACHN cells. The images were recorded at 0, 24, 48, and 72 h after scratching, where the black line indicates the front end of the migrating cells. (**D**) Representative images were taken from the Transwell assay, and box plots were counted for migrating and invading cells in each group with the magnification of 100X under the microscope. The 786-O cells and ACHN cells were selected as the *FoxP3*-silenced group and the normal control group, respectively. For the Transwell assay, the 4 groups of images on the left represent 2 types of cells grouped for migration and invasion, respectively. The dark-colored cells of the images are tumor cells. The right panel compares the number of migrating or invading cells in the two types of experimental groups with the control group, respectively.

To examine the effect of *FoxP3* on the biological processes in the entire network of genes, we performed high-throughput RNA sequencing to systematically analyze the changes in gene expression between the *FoxP3*-silenced 786-O cells and the controls. FDR < 0.05 and |log_2_FC| > 1 were screened out as significantly different genes and submitted to GSEA to run the hallmark gene sets. As shown in Figure 3A, there were “bumpy” enrichment plot gene sets in the control cells compared to the *FoxP3*-silenced 786-O cells (Figure 3A). Furthermore, we found that the gene sets “hallmark_myc_targets”, “hallmark_mTOR1_targets”, “hallmark_hypoxia_targets”, “hallmark_wnt_targets”, and “hallmark_E2F_targets” were responsible for 786-O control cells for the biological behavior of ccRCC. We also submitted TCGA RNA-seq data to GSEA to run the hallmark gene sets, which included two groups, *FoxP3* high-expression group (top 50) and *FoxP3* low-expression group (top 50) (Figure 3B). The results showed that the curve top of “hallmark_epithelia_mensenchymal_transition_targets” existed in the *FoxP3* high-expression group. Thus, higher *FoxP3* might be related to epithelial–mesenchymal transition (EMT) in ccRCC progression.

To verify the clinical sample bioinformatics analysis results, we performed *FoxP3* loss-of-function assays in 786-O and ACHN cell lines. As shown in Figure 3C, the wound healing assay results demonstrated that the *FoxP3* inactivation impaired the 786-O and ACHN cells’ migration by 60–80% at 72 h. We also applied a Transwell assay to assess the impact of *FoxP3* on the ccRCC migration capability (Figure 3D). The results further demonstrated that silencing *FoxP3* could significantly impair the 786-O and ACHN cells’ migration (786-O, *p* = 0.03; ACHN, *p* = 0.01) and invasion capabilities (786-O, *p* = 0.01; ACHN, *p* < 0.01). Our findings verified that a high expression of *FoxP3* could promote ccRCC cells’ migration and invasion in vitro.

### 2.3. FoxP3 Could Promote RCC Tumor Immune Evasion

**Figure 4:**
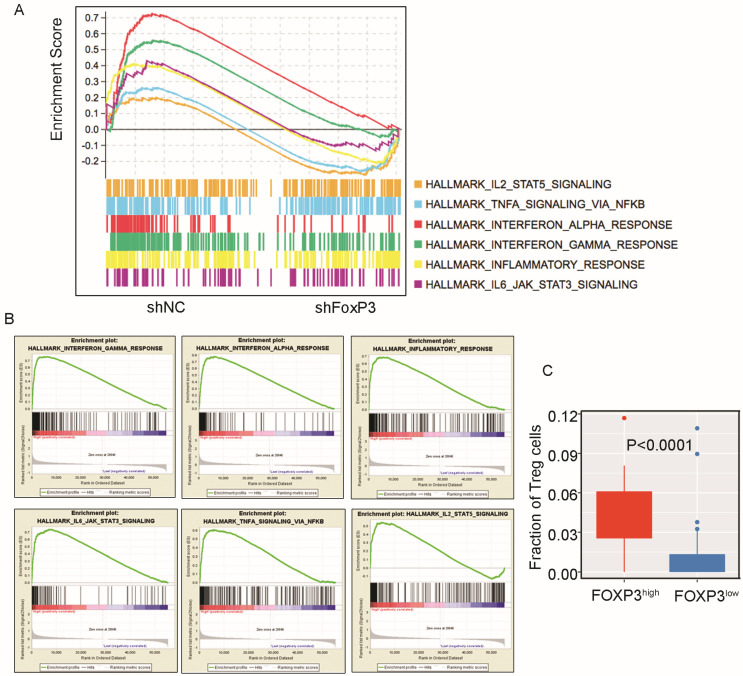
High expression of *FoxP3* could activate the expression of some immune-related pathways, which could induce Treg cells’ infiltration into the tumor microenvironment. (**A**) Gene Set Enrichment Analysis (GSEA) images showing the difference in expression of tumor-associated pathways between *FoxP3*-silenced 786-O cells and normal control 786-O cells. (**B**) Gene Set Enrichment Analysis (GSEA) images showing the difference in expression of each pathway between the *FoxP3* high-expression group (50 cases) and the *FoxP3* low-expression group (50 cases), respectively. (**C**) Box plots representing the distribution of fraction of Treg cells in the *FoxP3* high-expression group and the *FoxP3* low-expression group of ccRCC patients. Circles represent abnormal values within groups (*FoxP3*^high^: *FoxP3* high-expression group; *FoxP3*^low^: *FoxP3* low-expression group).

The immune function of *FoxP3* in Treg cells is clear, but the immune function of *FoxP3* in cancer is unclear, especially in RCC progression. To understand whether the *FoxP3* expression in tumor cells affects the RCC microenvironment, we analyzed the high-throughput RNA sequencing data in 786-O cells compared with silencing *FoxP3* in 786-O cells. The GSEA hall_mark_gene set results showed the immune-related signaling pathways “hallmark_IL2_stat5_signaling”, “hallmark_THFA_signaling_via_NFKB_targets”, “hallmark_inteferon_alpha_response_targets”, “hallmark_interferon_gamma_repsonse_targets”, “hallmark_inflammatory_response_targets”, and “hallmark_IL-6_JAK_stat3_response_signaling” (Figure 4A). In addition, the TCGA ccRCC data of the *FoxP3* high-expression group (top 50) and the *FoxP3* low-expression group (top 50) were sent to run hallmark gene sets. The results were consistent with our above data. “Hallmark_IL2_stat5_signaling”, “hallmark_inteferon_alpha_ response_targets”, “hallmark_interferon_gamma_repsonse_targets”, and “hallmark_ inflammatory_response_targets” were all enriched in the *FoxP3* high-expression group (Figure 4B). We also found that the patients in the *FoxP3* high-expression group had more Tregs infiltration in the tumor microenvironment (Figure 4C). Collectively, the results from our cell lines and TCGA ccRCC RNA-seq results indicated that *FoxP3* might play a pro-oncogenic role in ccRCC by activating immune-related pathways and by recruiting more Treg cells into the tumor microenvironment.

### 2.4. FoxP3 Silencing Inhibited RCC Growth in Mouse Xenograft Tumors

**Figure 5:**
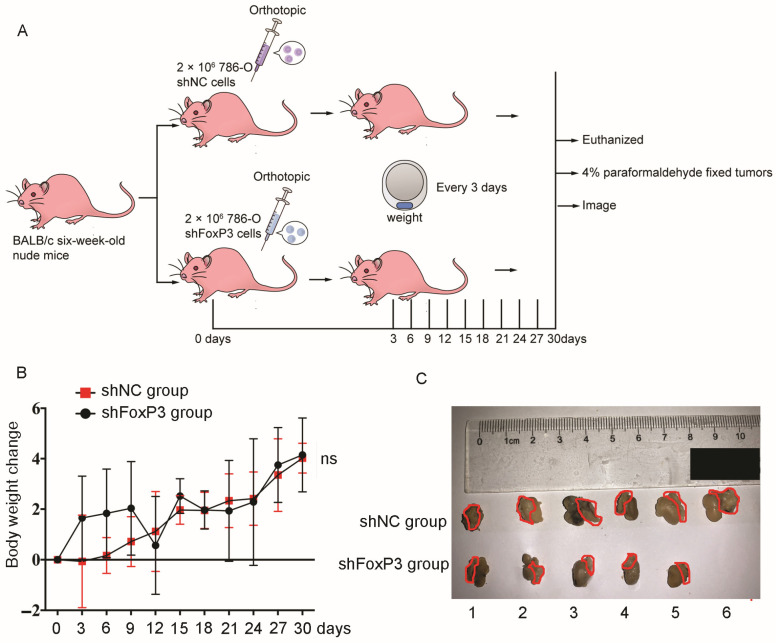
Orthotopic kidney cancer model experiments show that the inhibition of *FoxP3* expression could inhibit ccRCC growth. (**A**) Flow chart of the orthotopic kidney cancer model experiment. *BALB/c* nude mice were used, with 6 mice in each group. (**B**) The distribution of the body weight change of the 2 groups of mice in the experiment within 30 days after the injection. The horizontal line represents the time recorded after injection, and the vertical line represents the body weight change in the two groups, respectively (error bars indicate ± SD, *n* = 6; ns: no statistical significance). (**C**) Images of the kidneys together with tumor masses of nude mice in the two groups after the experiment. Nude mice were euthanized 30 days after injection. One nude mouse in the *FoxP3*-silenced group was euthanized early at 24 days after injection due to poor health status. The red circled area shows the tumor masses growing from the subcapsule of the kidneys of the nude mice.

For the *FoxP3* loss-of-function in vivo experiment, the 786-O cell line was selected in our study, because (1) 786-O is established as one of the first ccRCC cell lines and it has many characteristics of ccRCC; meanwhile, ccRCC is the most dominated pathological type among all RCC cases; (2) compared to other RCC cell lines, there was a higher expression level of *FoxP3* in the 786-O cell line; and (3) *SETD2* mutation could also be detected in the 786-O cell line. So, we constructed the 786-O orthotopic kidney cancer model to examine the effect of *FoxP3* in RCC in vivo. Two nude mouse groups were set up; one group (6 mice) was injected with 2 × 10^6^ cultured 786-O shNC cells and the other group (6 mice) was injected with 2 × 10^6^ cultured 786-O sh*FoxP3* cells into the subcapsule of both kidneys of each mouse, respectively (Figure 5A). The animals were euthanized at the end of 30 days (one mouse in the 786-O *FoxP3* KD group was euthanized at 24 days due to ill health). The two groups had similar rates of increase in weight (Figure 5B). However, the shNC group had a larger average tumor volume than the sh*FoxP3* group (Figure 5C). Collectively, sh*FoxP3* might inhibit RCC tumor mass growth in vivo.

## 3. Discussion

It is well known that RCC accounts for 3% of all cancers worldwide [2], and the current prognosis of patients with intermediate to advanced RCC is not very favorable [6,11]. Within the treatment and management options for patients with metastatic RCC, molecular targeted drugs and immune check-point inhibitor therapies have been heavily investigated. Previous studies have shown that Tregs are a class of cells that can regulate the body’s immunity [19] and may play different roles in tumors at different locations [31,34,35,36]; their biological functions can be regulated by the expression of *FoxP3* [15,16]. In recent years, it has been found that some normal tissues can also express *FoxP3* in addition to immune cells [30,31,32,33]. Therefore, exploring the expression and the biofunction of *FoxP3* in RCC tumor cells may help physicians to make better decisions in the treatment modalities. In our study, we constructed *FoxP3* in vivo and in vitro experiments, and we demonstrated that the expression level of *FoxP3* in ccRCC was increased by the induction of *BAP1* and *SETD2* mutation. The up-regulation of *FoxP3* could facilitate the migration and invasion of ccRCC cell lines, activate the immune-suppression-related pathways, and attract more Tregs infiltrated into the tumor microenvironment. All these would promote cancer cells immune evasion and metastasis, and eventually it could lead to worse outcomes for patients with ccRCC.

*FoxP3* is a key transcription factor in Tregs development and function and has been extensively studied [25,29]. In Tregs, the *FoxP3* promoter is regulated by forkhead box O protein 1 (*FOXO1*), forkhead box O protein 3 (*FOXO3*), and other regulatory elements [38,39]. *FoxP3* transcription is highly controlled by conserved noncoding sequences (CNSs), which can interact with some important transcription factors, such as human mothers against decapentaplegic homolog 3 (*SMAD3*), avian erythroblastosis virus E26 oncogene homolog 1(*ETS1*), *RUNX1*, *REL*, etc. [38,40]. However, the regulation of *FoxP3* in tumor cells is largely unclear, especially in RCC. In our study, we analyzed the *FoxP3* transcriptome across TCGA cancers, and we found that the *FoxP3* was highly expressed in the tumor tissues of ccRCC patients with *BAP1*- or *SETD2*-mutant genotype (Figure 1B). Then, we conducted an analysis in the CCLE database and found that the *FoxP3* expression was higher in RCC cell lines with *BAP1* or *SETD2* mutation than their wild-type cell lines (Figure 1F). In addition, we analyzed the staging of ccRCC with *FoxP3* in the GEO database and performed IHC in tissue microarrays from patients with ccRCC. The data showed that the *FoxP3* expression level was higher in tumors than in normal kidney tissues (Figure 1G and Figure 2A). This was similar to the study by Sell K et al., who performed RT-PCR on kidney tissue samples from RCC patients and found that the *FoxP3* expression levels in tumors were higher than those of adjacent normal kidney tissues [37]. However, unlike Sell K et al., who mainly focused on the aggregation of Tregs, our results might be associated not only with excessive Tregs but also with the overexpression of *FoxP3* in ccRCC tumor tissues caused by *BAP1*- or *SETD2*-mutant, and our findings might not have been reported yet. We also found that ccRCC tumors with a high stage could express higher levels of *FoxP3* than those with a low stage (Figure 2B–D), although no significant differences were found between different tumor grades. In general, we were able to observe that the high levels of *FoxP3* were often associated with poor tumor staging for patients with ccRCC. Interestingly, Hakimi AA et al. analyzed 188 ccRCC patients for genetic sequencing and prognosis and found that *BAP1* and *SETD2* mutations were associated with worse cancer-specific survival (CSS) [41]. A study of over 1000 ccRCC patients reached similar conclusions by Manley BJ et al., who found that ccRCC patients with mutations in *BAP1* and *SETD2* were associated with short CSS and recurrence-free survival, respectively [42]. These studies further validated our results that the poor prognostic outcome associated with mutations in *BAP1* and *SETD2* was likely to be related to the high expression level of *FoxP3*. In recent years, it has been found that the oncological behavior from the two genotypic mutations might be different, with previous studies suggesting that *SETD2* was associated with distant metastasis in ccRCC [43]. However, Peña-Llopis S et al. found that the *BAP1* mutation might promote tumor cells’ growth and make the prognosis of ccRCC patients even worse [44]. Although both gene mutation types resulted in the increased *FoxP3* expression in RCC, their different oncological behaviors still needed to be specifically analyzed by physicians when appropriate treatment strategies were considered. In summary, *FoxP3* was highly expressed in ccRCC with *BAP1*- or *SETD2*-mutant with advanced staging and could lead to poor prognosis for patients with ccRCC.

Most of the current studies are mainly focused on the effect of *FoxP3* in Treg cells in RCC. However, in our study, we also found that high expression of *FoxP3* in ccRCC cells was associated with patients’ poor prognosis. In gastric cancer, *FoxP3* could promote gastric cancer migration and invasion through the *TGF-β* pathway [35]. In breast cancer, *FoxP3* could induce the transcriptional activity of miR-200c and miR-141, which were elevated in patients with metastatic breast cancer [45]. In non-small cell lung cancer, *FoxP3* could promote tumor metastasis through the *Wnt*/*β*-*catenin* signaling pathway and EMT [36]. However, no report has described the *FoxP3* function in RCC progression. To explore the biological functions of *FoxP3*, we constructed the 786-O cells RNA sequencing experiment and analyzed the transcriptomic data. The GSEA showed that several gene sets of EMT-related genes were regulated by *FoxP3* (Figure 3A), and the “hallmark_epithelial_mesenchymal_transition” had a “bumpy” enrichment in the *FoxP3* high-expression group in the TCGA datasets (Figure 3B). Our wound healing assay and Transwell assay showed that *FoxP3* could promote RCC cells’ migration and invasion (Figure 3C,D). Consistent with our findings, several investigators have found similar results in different types of tumors. Previously, we mentioned that *FoxP3* has been shown to promote the EMT pathway in non-small cell lung cancer [36]. Wang L et al. found that increased *FoxP3* could lead to increased expression levels of miR-664a-3p, which might activate the EMT pathway and promote tumor progression in gastric cancer [46]. In addition, it has also been shown that *FoxP3* regulates the expression of LINC00885, and that high expression of *FoxP3* could promote cervical cancer cells’ proliferation and the activation of EMT pathways [47]. These results suggest that *FoxP3* could regulate different pathways of the EMT and promote cancer metastasis. Our study not only confirmed that *FoxP3* could activate the EMT pathway in ccRCC, but also provided related genes’ alteration by the high expression of *FoxP3*. Our results might indicate that we need to pay more attention to the high expression of *FoxP3* in RCC, because it could be one of the main factors leading to RCC metastasis.

Previous studies have demonstrated that increased *CD4*^+^*CD25*^+^*FoxP3*^+^ Treg cells in RCC were related to worse outcomes [33], and the *FoxP3*^+^ tumor cells have been detected in the tumor–normal tissue borders [37]. In our study, *FoxP3* could facilitate a tumor immune-suppressed microenvironment showed by 786-O RNA-sequence data and TCGA data (Figure 4A,B), and we found that more Treg cells could infiltrate into tumor tissues with an abundant expression of *FoxP3* (Figure 4C). However, in the study by Chakiryan et al., they analyzed the association between common somatic mutations in ccRCC and the tumor microenvironment, and they found that *SETD2* mutations were associated with significantly reduced levels of *FoxP3*^+^ T cells in tumors, stroma, and the tumor–stroma interface [48]. It has also been shown that the *SETD2*-mediated methylation pathway could inhibit IFN-α/β receptor signaling, which might ultimately impair the function of Tregs [49]. This result does not contradict our study; it might suggest that the *SETD2* mutations have a reduced ability to convene Tregs compared to other mutant phenotypes, and the results still need to be further explored. Our results were consistent with Hiroyuki et al.’s results, who demonstrated the *FoxP3* expression in non-small-cell lung cancer cells with tumor-infiltrating Tregs [50]. In pancreatic ductal adenocarcinoma, the *FoxP3* expression was demonstrated in pancreatic cancer cells with tumor-infiltrating Tregs through the *FoxP3*/C-C motif chemokine ligand 5 (*CCL5*)/C-C motif chemokine receptor 5 (*CCR5*) pathway [27]. In murine melanoma, it was demonstrated that *FoxP3* shifted the environment toward an immunosuppressive response by modifying the immune system [51]. These findings are quite similar to our results, suggesting that high expression of *FoxP3* could activate immune-related pathways, recruit more Treg cells’ infiltration into the tumor microenvironment, and eventually promote immune evasion for cancer cells. In addition, our nude mouse orthotopic kidney cancer experiments showed that the growth of ccRCC cells with *FoxP3* knocked out was significantly inhibited compared to controls (Figure 5C). This result further suggests the role of *FoxP3* for ccRCC growth in vivo.

There are very few studies on the effect of *FoxP3* produced by RCC cells themselves on their growth, and more studies have explored the effect of Tregs on RCC. Tregs can suppress the body’s immune response [19]; for example, Liotta F et al. found that Tregs exhibited inhibitory activity to effector T cells isolated from kidney tumors in vitro [52]. Ning et al. found that increased Tregs in tumor infiltration were positively correlated with *VEGF* protein expression [53]. In contrast, our study firstly proposed the relevant genes causing increased *FoxP3* expression in RCC and identified possible pro-growth pathways and immune escape pathways induced by *FoxP3* expression in RCC cells. These pathways demonstrated a novel role for *FoxP3* in RCC growth, and might be used to further elucidate the effect of immunopharmaceutical treatment; they could also help in the development of other treatment regimens. The main limitations for our study were that most of our experiments were conducted in vitro, and that there was a lack of sufficient clinical data from RCC patients and a lack of study of detailed mechanisms to demonstrate that the *BAP1*- or *SETD2*-mutant could indeed activate the specific growth, metastatic, and immune escape signaling pathways in RCC compared to other genotypic mutations. In the future, with the development of studies for novel genes, such as *FoxP3*, and their related regulatory mechanisms in RCC progression exploring a more precise and effective individualized therapy might be a possible direction.

## 4. Materials and Methods

### 4.1. Patient Samples and Bio-Information Analysis

*FoxP3* transcriptome (count and FPKM value) across TCGA cancers (The Cancer Genome Atlas) data were analyzed on the website (http://gepia2.cancer-pku.cn/#index, accessed on 6 June 2021). The signature score was calculated by mean value of log2 (TPM + 1) of *FoxP3* gene in gene set. The red box indicated the tumor samples while the blue one represented the normal tissues [54]. The effect of the *FoxP3* expression on the overall survival (OS) and disease-free survival (DFS) was determined in UALCAN, which is an interactive web resource using TCGA datasets to analyze data from cancer patients. The mRNA expression of *FoxP3* in ccRCC of different stages/grades was determined online using the UALCAN website (http://ualcan.path.uab.edu, accessed on 15 August 2021). Individual cancer stages were based on AJCC (American Joint Committee on Cancer) pathologic tumor stage information, and samples were divided into stage I, stage II, stage III, and stage IV groups. Tumor grade information was available in the TCGA database, and samples were categorized into grade 1, grade 2, grade 3, and grade 4 groups. Using the CPAN module “Statistics::Descriptive”, mean TPM values (10 or above were retained) of each gene in normal samples and tumor samples were obtained separately [55]. The GSE781 dataset in the GEO database (Gene Expression Omnibus, https://www.ncbi.nlm.nih.gov/geo/, accessed on 18 October 2021) was accessed to examine *FoxP3* expression in ccRCC tumor and normal tissues. The *FoxP3* expression with relationships to *PBRM1*^MT^, *VHL*^MT^, *SETD2*^MT^, and *BAP1*^MT^ in ccRCC was analyzed using TCGA KIRC transcriptome (count and FPKM value) and mutational data. In the analysis, we first removed the low-value genes using a heterogeneity analysis; then, we normalized the data sets using the variance stabilizing transformation (VST) method in the DESeq2 package, as previously described [56].

### 4.2. CCLE Analysis in RCC Cell Lines

The *FoxP3* RNA expression in RCC cell lines was determined by CCLE (Cancer Cell Line Encyclopedia (https://portals.broadinstitute.org/ccle/home, accessed on 10 November 2021), including 1000 cell lines’ gene expression, DNA copy numbers, histone profiling, RNA-seq, and DNA methylation from more than 20 cancer types [57]. The gene mutation information of *VHL*, *PBRM1*, *BAP1*, and *SETD2* in ccRCC cell lines was applied according to Wei X et al.’s article [7].

### 4.3. Gene Set Enrichment Analyses

We downloaded 611 ccRCC patients’ RNA-seq data from the TCGA database. For the Gene Set Enrichment Analysis (GSEA), the *FoxP3* high-expression group (top 135 (25% of 611)) and *FoxP3* low-expression group (top 134 (25% of 611)) were set up in our study. *FoxP3* Text.gct and *FoxP3* text.cls were submitted to GSEA 4.1.0 version, and the hallmark gene sets were selected for the analysis.

### 4.4. Cell Culture

The 786-O and ACHN cells were purchased from American Type Culture Collection (ATCC, https://www.atcc.org, accessed on 5 December 2021) and authenticated by STR profiling in one year. The 786-O and ACHN cells were cultured with an RPMI medium (Cytiva, Shanghai, China) with 10% fetal bovine serum (Biological Industries, Kibbutz Beit Haemek, Israel) at 37 °C in a humidified 5% CO_2_ incubator.

### 4.5. Plasmids and Transfection

pGPu6/Neo is a plasmid vector that encodes short hairpin RNA (shRNA) that targets FoxP3 or scrambles (shNC). It was constructed by GenePharma Inc. (Shanghai, China). The plasmid was transfected into 786-O and ACHN cells using the Roche X-tremeGENE DNA transfection reagent (Roche Co., Ltd., Shanghai, China) according to the manufacturer’s instructions. After 48 h of transfection, real-time quantitative PCR and Western blotting were used to verify the *FoxP3* mRNA and protein expression, respectively. The targeting sequence of *FoxP3* was as follows:

sh*FoxP3*-1: 5′-GATCCAAAAAAGGACCATCTTCTGGATGAGAATCTCTTG AATTCTCATCCAGAAGATGGTCC-3′;

sh*FoxP3*-2: 5′-GATCCAAAAAAGTCTGCACAAGTGCTTTGTGCTCTCTTG AAGACACAAAGCACTTGTGCAGAC-3′;

sh*FoxP3*-3: 5′-GATCCAAAAAAGCCATGGAAACAGCACATTCCTCTCTTG AAGGAATGTGCTGTTTCCATGGC-3′;

shNC: 5′-GATCCAAAAAATTCTCCGAACGTGTCACGTTCTCTTGAAACG TGACACGTTCGGAGAAC-3′.

### 4.6. Immunohistochemistry

Tissue microarrays (#HKidE180Su03) were purchased from Shanghai Outdo Biotech Co., Ltd. (Shanghai, China). HKidE180Su03 is a 180-spot, paraffin-embedded tissue array including 90 paired ccRCC patients with 5 years of follow-up data. As previously described [58], immunohistochemical staining (IHC) of *FoxP3* (Abcam, Boston, MA, USA; #ab196022, dilution 1:200) was performed using a DAKO Autostainer Plus system (#GK600505, Gene Tech Company, Shanghai, China). The IHC score was calculated by multiplying the intensity and percentage scores. IHC staining was graded as follows: 0 for 0%, 1 for ≤25%, 2 for 25–50%, 3 for 50–75%, and 4 for ≥75%. The IHC intensity was scored as follows: 0 for no staining, 1 for weakly positive staining, 2 for moderately positive staining, and 3 for strongly positive staining.

### 4.7. Quantitative RT-PCR

After 48 h of sh*FoxP3* plasmid transfection, the total RNA from cells was isolated using the RNA fast 200 kit (Feijie Biotech, Shanghai, China) and reverse-transcribed using the Prime Script™ RT reagent kit (Takara Biotechnology Co., Ltd., Dalian, China). A CFX96 Real-Time PCR system (Bio-Rad, Hercules, CA, USA) and SYBR Green PCR Master Mix (Takara Biotechnology Co. Ltd., Dalian, China) were applied for the gene expression. The relative gene expression was calculated by the 2^−ΔΔCt^ method using 18S as a reference gene. The sequences of the 18S and *FoxP3* primers were as follows:

18S forward, 5′-ATGGGGAAGGTGAAGGTCGG-3′;

18S reverse, 5′-GACGGTGCCATGGAATTTGC-3′;

*FoxP3* forward, 5′-GTGGCCCGGATGTGAGAAG-3′;

*FoxP3* reverse, 5′-GGAGCCCTTGTCGGATGATG-3′.

### 4.8. Western Blot Analysis

The cell lysis and Western blotting protocols were previously described. Thirty micrograms of whole protein was separated by 10% SDS-PAGE (#PG113, Epizyme, Shanghai, China). An anti-*FoxP3* antibody (Cell Signaling Technology, Shanghai, China; #12632, dilution 1:1000) and anti-*β-actin* antibody (Abclonal, Wuhan, China; #AC004, dilution 1:1000) were used. The secondary antibodies were anti-mouse IgG (Beijing Zhongshan, Beijing, China; #ZB-2305, dilution 1:2000) and anti-rabbit IgG (Beijing Zhongshan, Beijing, China; #ZB-2301, 1:2000).

### 4.9. Wound Healing Assay

The wound healing assay was performed as previously described [59]. After 48 h of sh*FoxP3* plasmid transfection, 786-O and ACHN cells were seeded on 6-well plates and reached 100% confluence. The cells were starved overnight, and a wound was made by using a sterile 200 μL pipette tip to scratch the artificial wounds. The cells were washed with PBS 3 times. Wound healing was observed by microscopy after 24, 48, and 72 h.

### 4.10. Transwell Assay

The 786-O and ACHN cells were harvested after 48 h of sh*FoxP3* plasmid transfection. Then, 5 × 10^4^ cells in 200 μL serum-free RPMI 1640 were added into the upper chambers containing 8 μM pore polycarbonate membrane filters (Millipore, Burlington, MA, USA). For the invasion assay, 5 × 10^4^ cells in 200 μL serum-free RPMI 1640 were added into the upper chamber inserts with Matrigel (BD Biosciences, Franklin Lakes, NJ, USA), which had been plated 4 h in advance. Then, 800 μL RPMI 1640 medium containing 10% FBS was added to the lower chambers. After 24 h, the Transwell inserts were fixed with 4% paraformaldehyde for 10 min and stained with 0.1% crystal violet for 15 min at room temperature. Then, the migrating and invading cells were captured and counted under a light microscope.

### 4.11. High-Throughput RNA Sequencing

*FoxP3* knockdown (*FoxP3*-silenced 786-O cells) and control cells (shNC transfected 786-O cells) lysates were collected and sent to GENE DENOVO (Guangzhou, China) for high-throughput RNA sequencing.

### 4.12. Animal Experiments

Twelve *BALB/c* nude mice (6 weeks old, male) were randomly separated into two groups and injected with 2 × 10^6^ 786-O cells (control or sh*FoxP3* cells) into the subcapsule of both kidneys of each mouse. Our animal experiments were conducted under the Animal Research: Reporting of In Vivo Experiments (ARRIVE) guidelines and approved by the institutional review board of the First Affiliated Hospital of Xi’an Jiaotong University. The animals were euthanized on the 30th day or when the mice showed clear signs of ill health. The animal weights were measured every 3 days for 30 days.

### 4.13. Statistical Analysis

Each experiment was repeated 3 times. Differences between two groups (Student’s *t*-test) were compared using the GraphPad Prism software (Version 6.0 software, GraphPad, Boston, MA, USA), and data are shown as the mean ± SD with error bars. *p* < 0.05 was considered statistically significant in our study.

## 5. Conclusions

Our study demonstrates that *FoxP3* was increased in RCC cells with *BAP1*- or *SETD2*-mutant. Increased *FoxP3* could not only activate the EMT pathway in RCC tumors, but also induce the immunosuppressive microenvironment of tumors and eventually attract more Tregs cells to activate RCC growth and metastasis. Approaches to investigating the specific mechanism of *FoxP3* in RCC progression should be explored in the future [60].

## Data Availability

Data are contained within the manuscript. Experimental raw data supporting the reported results are available from the corresponding author upon request.
